# Tissue-Specific Variations in Transcription Factors Elucidate Complex Immune System Regulation

**DOI:** 10.3390/genes13050929

**Published:** 2022-05-23

**Authors:** Hengwei Lu, Yi-Ching Tang, Assaf Gottlieb

**Affiliations:** Center for precision health, School of Biomedical Informatics, University of Texas Health Science Center at Houston, Houston, TX 77030, USA; hengwei.lu@uth.tmc.edu (H.L.); yi-ching.tang@uth.tmc.edu (Y.-C.T.)

**Keywords:** transcriptional regulation, transcriptome imputation, transcription factor polymorphism

## Abstract

Gene expression plays a key role in health and disease. Estimating the genetic components underlying gene expression can thus help understand disease etiology. Polygenic models termed “transcriptome imputation” are used to estimate the genetic component of gene expression, but these models typically consider only the *cis* regions of the gene. However, these *cis*-based models miss large variability in expression for multiple genes. Transcription factors (TFs) that regulate gene expression are natural candidates for looking for additional sources of the missing variability. We developed a hypothesis-driven approach to identify second-tier regulation by variability in TFs. Our approach tested two models representing possible mechanisms by which variations in TFs can affect gene expression: variability in the expression of the TF and genetic variants within the TF that may affect the binding affinity of the TF to the TF-binding site. We tested our TF models in whole blood and skeletal muscle tissues and identified TF variability that can partially explain missing gene expression for 1035 genes, 76% of which explains more than the *cis*-based models. While the discovered regulation patterns were tissue-specific, they were both enriched for immune system functionality, elucidating complex regulation patterns. Our hypothesis-driven approach is useful for identifying tissue-specific genetic regulation patterns involving variations in TF expression or binding.

## 1. Introduction

Transcription regulation plays a critical role in cellular states, while transcriptional misregulation has been associated with a broad range of diseases [1]. Thus, the identification of genetic components that help explain gene transcription levels is valuable in order to generate accurate models that can further associate variations in gene expression with specific diseases. This is especially beneficial in tissues that are not readily accessible for direct measurement of gene expression.

Previously published methods for identifying gene regulation include expression quantitative trait loci (eQTLs) [2] and, more recently, transcriptome imputation techniques, such as PrediXcan or TWAS [3,4], which models the genetic component of observed gene expression using combinations of genetic locations. While eQTLs focus on single variants with strong effect size, transcriptome imputation techniques demonstrate that combinations of (possibly) smaller-effect genetic variants can explain transcription levels better.

Transcriptome imputation methods typically include only variants in *cis* with the gene they impute, owing in part to the large space of possible trans-acting locations. For many genes, however, these *cis* models explain only a small portion of the variability in gene expression. Indeed, a paper by Liu et al. [5] estimated that most heritability is driven by weak trans-eQTL single-nucleotide polymorphism (SNPs), supporting an approach to identify such sources of expression variability.

Despite the large space of trans-acting loci, a method by Wheeler et al. [6] used a data-driven approach to identify correlated trans-acting genes based on the PrediXcan transcriptome imputation framework. They identified 55 significant associations between the expression of trans-genes and the imputed expression of 39 genes in blood tissue. However, their data-driven approach was limited to pairwise trans-gene correlations, required a strict false-discovery threshold rate due to the large hypothesis space, and, most importantly, the models lacked suggested mechanisms that limited the biological interpretation of the discovered correlations. Despite these limitations, Wheeler et al. found that trans-genes were enriched with transcription factor (TF) pathways, and target genes were enriched in transcription factor binding sites, supporting a hypothesis-driven approach that focuses on TFs as a source of expression variability.

Here, we develop a hypothesis-driven approach to identify associations between variations in TFs and variation in the transcription levels of their transcribed genes, suggesting a higher level of regulation. While regulation through polymorphism in TF-binding sites is well documented, as TFs regulate transcription by binding to *cis*-regulatory regions in the vicinity of the regulated genes [7], polymorphism in TFs themselves has been previously associated with diseases such as hypertension, coronary artery disease, type 2 diabetes mellitus, and dyslipidemia [8,9,10]. Thus, we hypothesize that variability in TFs may indirectly mediate an effect on disease phenotypes through their effect on the transcription levels of their transcribed genes. This approach addresses previous limitations in identifying trans-genes, since it provides a model that looks at the combinatorial effects of all the TFs affecting transcription of a gene instead of a single trans-gene, reduces the multiple hypotheses space, and provides a mechanistic interpretation of these trans-models.

We considered two possible mechanisms where variability in a TF can lead to variability in the expression of a gene: one mechanism considers variations in the expression of TFs to correlate with variations in the expression of their transcribed genes. Another mechanism suggests that non-synonymous single-nucleotide polymorphisms (SNPs) within TFs with predicted deleterious effects may affect the binding affinity and thus the transcription levels of the transcribed genes. We thus developed two distinct models to capture these potential mechanisms. With these models, we are able to account for transcriptional variability that is only partially explained by *cis* imputation models and suggest potential biological mechanisms for this higher-level regulation. We applied our models to two tissues, skeletal muscle and whole blood, identifying 1035 hit genes where the TF model can explain a significant portion of the variability beyond the *cis* models, with 76% explaining more variability than the *cis* models alone.

To summarize, our hypothesis-driven approach introduces three contributions: (1) we detect cumulative weak effects that individual trans-eQTLs may have insufficient power to detect; (2) our hypothesis-driven approach is biologically interpretable; and (3) it reduces the number of hypotheses relative to data-driven approaches. Thus, our models enable the identification of higher-level regularization of TFs, which may further suggest clinical implications through their effect on disease-associated genes.

## 2. Materials and Methods

### 2.1. Data

Genotype and expression data from the Genotype-Tissue Expression Project (GTEx) version 8 [11] were retrieved from dbGaP. Transcription factors and their transcribed genes (204,999 unique gene–TF pairs, see Table 1 for statistics) were assembled from three sources: Transcriptional Regulatory Relationships Unraveled by Sentence-based Text mining (TRRUST V2) [12], the Human Transcriptional Regulation Interaction Database (HTRIdb) [13], and the Regulatory Network Repository of Transcription Factor and microRNA Mediated Gene Regulations (RegNetwork) [14]. Genomic positions of the TFs were computed based on the Human Genome Assembly version 37 (GRCh37). Functional annotations for non-synonymous SNPs were retrieved from SnpEff v4.3 [15].

### 2.2. Constructing TF Models

**Figure 1:**
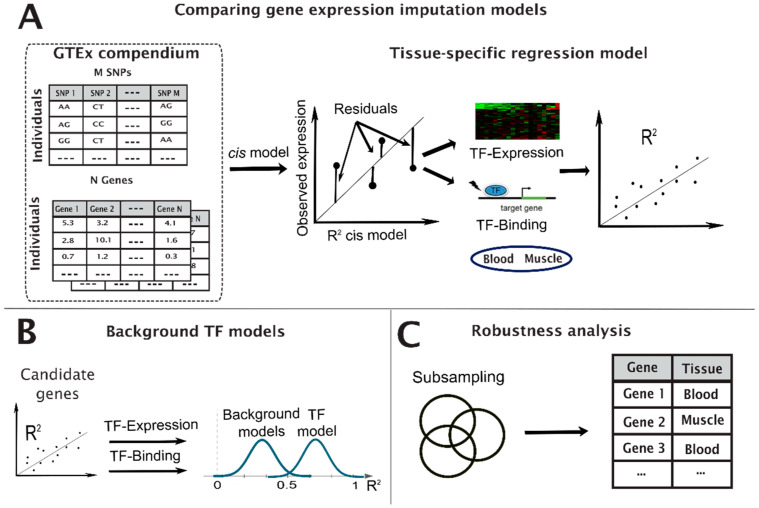
Illustration of the filtering pipeline to identify TF hit genes. We computed the baseline *cis* model and compared them with one of the three TF models to identify candidate genes per TF model (**A**), computed a background model for each of the candidate genes to test their significance (**B**), and conducted a robustness test to validate the results (**C**).

The constructed TF models follow a five-step protocol (Figure 1).

Step 1, normalized observed genes expression: We followed the PrediXcan recommended normalization to retain only the genetic component of the observed expression by accounting for gender, sequencing platform, the top 3 principal components of the genotype dosages, and the 15 probabilistic estimation of expression residuals (PEER) factors [16] for each gene’s observed expression. The residuals between the linear model formed by these factors and the observed expression values from GTEx are the normalized gene expression considered in the following steps.

Step 2, apply PrediXcan: We applied the pre-trained *cis* models (baseline) developed in the PrediXcan method [16], coded in Python and R. PrediXcan methods predict the expression values of each gene in the selected tissue based on their genotypes.

Step 3, build a model for candidate genes: We modeled the residuals between the normalized observed expression and the PrediXcan model of the genes using regularized LASSO regression [17]. The independent variables for the TF-expression model were the expression of the TFs that are associated with each candidate gene and, for the TF-binding model, the dosages of a set of non-synonymous SNPs within these TFs. In order to focus on the SNPs most likely to affect TF binding, we retained only the non-synonymous SNPs that are considered deleterious according to SIFT [18] (SIFT score < 0.05).

Step 4, background models: We filtered the candidate genes based on significance in two background models: The first background model shuffled the residuals and re-trained the models one hundred times. For the second filter, we evaluated the significance of each candidate gene relative to the random selection of non-associated TFs. We selected 100 random sets of TFs for each candidate gene (each set with the same number of TFs as the true set of TFs associated with the gene) and computed the R^2^ for each random set of TFs. We retained only genes that passed both background models with significance level of 0.05 (adjusted *p*-value using Benjamini Hochberg false-discovery rate [19]).

Step 5, robustness check: A robustness check was conducted by randomly removing 10% of the samples and re-running the pipeline. Genes that were discovered in more than 50% of ten robustness runs (i.e., at least five of the runs) were declared hit genes.

### 2.3. Statistical Analysis

Statistical significance of overlap with other works was computed using the hypergeometric test. Enrichment of hit genes with biological functions was performed based on the hypergeometric test using the ToppGene suit [20].

## 3. Results

### 3.1. Identifying Gene Expression Associated with TF Variability

**Figure 2:**
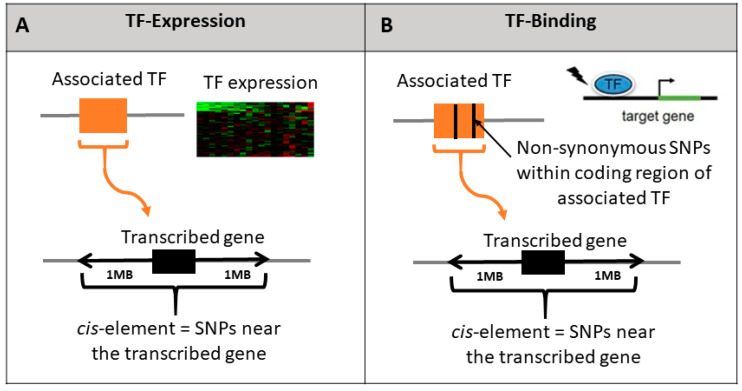
Illustration of the three tested hypotheses regarding the effect of TF genetic polymorphism on the expression of their transcribed genes. We test whether polymorphism in the TF (orange box) affects the transcription levels of the transcribed gene (black box) by testing the added effect of each TF model relative to the baseline *cis* model. TF-expression model includes association of the TF expression with imputed gene expression (**A**); TF-binding model includes non-synonymous SNPs within the associated TF boundary (**B**).

We hypothesized that variability in TFs may be associated with variability in the expression of their transcribed genes. We considered two potential mechanisms to test this hypothesis: (1) variability in transcription levels of the TF that subsequently translate to variability in the transcription levels of the transcribed gene (Figure 2A); or (2) SNPs within TFs can affect change in binding affinity of the TF to the *cis*-binding sites of their transcribed genes (Figure 2B). We refer to these models as TF-expression and TF-binding, respectively (Section 2, Figure 1).

We developed a pipeline that tests model significance and robustness with two background models and a robustness test to reduce false positives (Section 2). To demonstrate our method, we focused on the two tissues with the largest sample size in the genome tissue expression (GTEx) compendium: skeletal muscle and whole blood. Table 1 provides sample sizes and statistics for the two tissues.

We term “hit genes” as genes where the model (TF-expression or TF-binding) provided a significant improvement in explaining the gene transcription levels over the base *cis* model, passing all significance tests (Section 2, Figure 1). Following these tests, we identified 1095 hit genes (1035 unique), with 379 and 419 hit genes from the TF-expression model in muscle and blood tissues, respectively, and 155 and 138 hit genes from the TF-binding model on muscle and whole-blood tissues, respectively (Tables S1 and S2). Forty-one of the hit genes (4%) were discovered in both tissues by the TF-expression model, while none were shared between tissue in the TF-binding models, suggesting it is more tissue-specific.

Notably, the TF-expression models were more robust to the robustness test, with only 6 genes in skeletal muscle and 17 in blood that did not pass this filter, while in the TF-binding models, 183 genes in muscle and 167 genes in blood that did not pass the robustness test (see Section 2). Only one gene from muscle shares SNPs used in the *cis* model with the TF SNPs used in our TF-binding model, and none are shared in blood.

**Figure 3:**
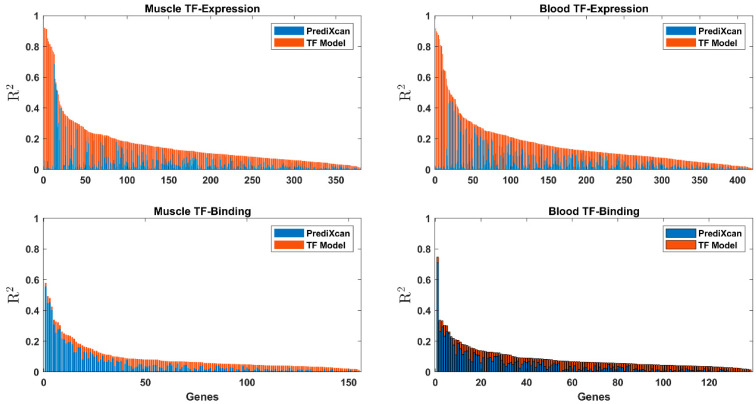
Stacked bar graph of the R^2^ results of the *cis* baseline model (blue) and the skeletal muscle and whole-blood TF-expression and TF-binding (orange).

There was a low correlation between the *cis*-based R^2^ and R^2^ of the TF-expression models (Pearson ρ ranged between −0.12 and 0.06, with significant *p*-values after correction for false-discovery rate only for blood TF-expression, see also Figure 3), suggesting that the *cis* and trans effects are generally independent.

### 3.2. Comparison with Other Methods

We first compared the large-scale trans-eQTLs dataset of Võsa et al. [21]. They identified 3966 genes with trans-eQTLs in whole blood, 2909 of which were tested in our whole-blood set. While they reported very low reproducibility in GTEx (between 0.07% and 0.09%), we still found that 175 (of the 419 hit genes, 42%) discovered in whole-blood TF-expression also had trans-eQTLs discovered in their whole-blood sample (hypergeometric *p*-value < 10^−10^). Additionally, 30 out of 138 genes (22%) were discovered through the TF-binding model in blood, but this overlap is not statistically significant. While the eQTLs of Võsa et al. [21] were discovered in whole-blood tissue, we still found significant overlap with genes discovered with our TF-expression model within muscle tissue (128/379, *p* < 3 × 10^−6^). This overlap was not significant in muscle TF-binding (45/155 shared genes).

One gene out of seven from the dataset of Võsa et al. [21] that were replicated in GTEx whole-blood tissue, hemoglobin subunit γ 1 or γ-globin (HBG), overlapped with our hit genes in the blood TF-expression model, but the overlap was not statistically significant. Similarly, one gene, ARL6IP1, was replicated in GTEx skeletal muscle and also detected by our skeletal muscle TF-expression model (hypergeometric test *p* < 0.03). Both of these genes had a larger trans effect (R^2^ = 0.04 and 0.13 in blood and muscle, respectively) than the *cis* model (R^2^ = 0.008 and 0.08). We highlight these two genes below.

The highest-weighted TF in the TF-expression model of whole blood for HBG1 is Krüppel-like factor 3 (KLF3) (weight = −0.13). KLF3 represses a subset of KLF1/EKLF target genes and is required for proper erythroid (cells differentiated from hematopoietic stem cells) maturation [22]. KLF1 in human and mouse adult erythroid progenitors markedly increases human γ-globin expression ratios [23], while EKLF protein activates γ-globin in the fetal-like erythroleukemia cell line [24]. Interestingly, previous evidence shows that HBG1, KLF1, and KLF3 are up-regulated in the β globin knockout human erythroid progenitor cell model, relative to controls [25] and in human myeloid leukemia cells [26]. Finally, other TFs in the model are NF-YA (weight = 0.11), GATA-2 (weight = −0.08), and GATA-1 (weight = 0.04), where NF-Y recruits both the transcription activator and repressor to modulate the tissue-specific expression of the human γ-globin gene, with GATA-2 part of the transcription activator hub and GATA-1 as part of the transcription repressor hub [27,28,29].

Mutations in ARL6IP1 are associated with spastic paraplegia 61, a rare inherited disorder that causes weakness and stiffness in the leg muscles [30,31]. The TFs with the highest weight in the model for ARL6IP1 are GATA-2, GATA-3, and PPARG (weights = 0.11, 0.13, and 0.14, respectively), all with their reported association with skeletal muscle tissue. Specifically, GATA-2 and GATA-3 are expressed in skeletal muscle cells [32], where GATA-2 is a unique marker of hypertrophy in skeletal muscle fibers [33,34], and GATA-3 promotes the invasion of tumors into skeletal muscles [35]. Additionally, PPARG inhibits NFKB-dependent transcriptional activation in skeletal muscle [36], and PPARG polymorphism is associated with improved glucose utilization in skeletal muscles [37]. Interestingly, the trans-eQTLs associated with ARL6IP1 (rs6592965 and rs12718598) both reside in the intron of the TF gene IKZF1. While IKZF1 does not bind directly to ARL6IP1, GATA-3 is up-regulated in IKZF1-deleted samples [38], suggesting potential co-regulation between the TFs.

Our second comparison was with the results of Wheeler et al. [6], who used a data-driven approach to test the association of the expression of trans-acting genes with the imputed expression of other genes (via PrediXcan). They identified 39 genes with trans associations (22 and 33 of which are part of our initial gene set of muscle and blood tissues, respectively). Seven of these genes were predicted also by our pipeline, with five of these shared genes identified in whole blood (CRISP3, NBL1, ASTL, and MANSC1 in blood TF-expression and ZNF135 in blood TF-binding; hypergeometric probability, *p* < 0.03) and two, CLEC12A and RNASE6, in muscle tissue TF-expression (but not statistically significant). This corresponds well with our expectations, as Wheeler’s genes were discovered in whole-blood tissue.

Out of these seven genes shared with Wheeler et al., we highlight the two genes with the highest model R^2^ in blood (cysteine-rich secreted protein 3 (CRISP3)) and ribonuclease A family member K6 (RNASE6)) in muscle tissue. CRISP3 is activated in mouse B cells and may be a potential biomarker of multiple myeloma [39,40]. The TF with the largest weight in the model is the ETS-related gene (ERG), which is shown to be essential for early B lymphoid differentiation [41] and nuclear expression of ERG in a vast majority of myeloma cells [42]. Furthermore, increased expression of ERG is indicative of poor prognosis of acute lymphoblastic leukemia and cytogenetically normal acute myeloid leukemia [43].

The second gene shared with Wheeler et al. is RNASE6, showing a substantial increase in explained variability (R^2^ = 0.18, while PrediXcan R^2^ = 0.02) in muscle tissue. Spi-1 proto-oncogene (SPI-1) is the TF with the highest weight in the TF-expression model of RNASE6. The detected genes and their TF are associated with skeletal muscle [44,45,46].

### 3.3. Hit Genes in Expression Models Are Enriched with Immune Response

The TF-expression models were highly enriched in immune system Gene Ontology biological processes, including top biological processes of activation of immune cells, including leukocyte, myeloid cells, neutrophil, and granulocyte activation involved in immune response in muscle tissue (Benjamini–Hochberg (B&H) FDR-adjusted *q*-value < 3 × 10^−17^) and immune system development, regulation of the immune system, and lymphocyte and leukocyte proliferation in blood tissue (B&H FDR-corrected *q*-value < 8 × 10^−7^), as well as blood-specific terms such as hemopoiesis (*q*-value < 2 × 10^−7^) and a human phenotype of hemolytic anemia (*q*-value < 2 × 10^−3^) (Table S2). Additionally, genes discovered in both TF-expression model tissues are enriched for the cellular location of the major histocompatibility complex (MHC) protein complex (B&H FDR-adjusted *q*-value < 3 × 10^−6^ and *q*-value < 2 × 10^−3^ for muscle and blood, respectively). MHC class II is known to be expressed in skeletal muscle [47,48] and is tightly regulated by a variety of transcription factors [49,50].

The TF-expression model in both tissues was also enriched for the autoimmune disease systemic lupus erythematosus (SLE) (with 76 and 65 associated genes and FDR-adjusted *q*-value < 6 × 10^−15^ and *p* < 3 × 10^−9^ for muscle and blood tissues, respectively). Indeed, SLE patients have a high prevalence of leukopenia, lymphopenia, and neutropenia [51], as well as inflammatory myositis [52]. Additionally, greater skeletal muscle mitochondrial dysfunction is found among fatigued patients with SLE [53].

In contrast to the TF-expression model, the TF-binding model in both tissues has no enriched Gene Ontology biological processes. TF-Binding in muscle is enriched for coated vesicle membrane and for the cellular compartment of ER to the Golgi transport vesicle membrane (*q*-value < 0.02). Indeed, extracellular vesicles have a role in skeletal muscle [54,55]. During skeletal muscle differentiation, the Golgi complex undergoes dramatic reorganization [56], and muscle impulse activity plays a major role in regulating the organization of the Golgi complex in the extra-junctional region of muscle fibers [57]. We highlight two Golgi-related hit genes in muscle TF-binding. The first is Bet1 Golgi vesicular membrane trafficking protein (BET1), where low BET1 protein levels are associated with impaired ER-to-Golgi transport in congenital muscular dystrophy [58]. The second example is NOTCH2, where Notch signaling plays a critical role in the regulation of embryonic and post-natal skeletal myogenesis, and one of its activation stages is mediated in the Golgi apparatus [59].

As evident from the low overlap between tissues and models, the discovered genes were tissue- and mechanism-specific, suggesting fine-tuned regulation (Tables S1 and S3). The exceptions are four hit genes (MORN1, CYP11B1, FAM222A, and FAM91A1), discovered by both TF models in blood tissue, and five genes (HLA-DRB1, NCF2, SNX20, SIAE, and BCHE), discovered in muscle tissue by both models.

We highlight the major histocompatibility complex, class II, DR β 1 (HLA-DRB1), which was discovered in both tissues in muscle tissue by both models. Our model explains the same amount of variability of HLA-DRB1 as the *cis* model in both tissues. PrediXcan obtained R^2^ = 0.16 and R^2^ = 0.11 on blood and muscle tissues, respectively, and our TF-expression model obtained the same in each tissue on top of the PrediXcan model. The TF-binding model obtained a smaller R^2^ of 0.02 in the muscle tissue (Table S1, Figure 3). Indeed, it was previously reported that both *cis*- and trans-regulatory polymorphisms modulate the expression of HLA-DRB1 [60,61].

The connection between variations in HLA-DRB1 and its TFs is further supported by reported associations between the expression of HLA-DRB1 and the expression of its TFs with the same disease. Specifically, the TF with the highest weight in the TF-expression model (β = 0.23 in muscle and β = 0.18 in blood) is CIITA, where variability in the CIITA gene has been reported to interact with specific mutations of HLA-DRB1 to increase the risk for multiple sclerosis [62,63] and type 1 diabetes [64,65]. Additionally, the expression of HLA-DRB1 is also predictive of type 2 diabetes [66], as is the expression of CIITA and another HLA-DRB1 TF, RFX5, which determine susceptibility to type 2 diabetes mellitus [67]. In relation to the specificity of the tissues, skeletal muscle insulin resistance is the primary defect in type 2 diabetes [68,69]. While the TF-binding model explains lower variability in HLA-DRB1 than TF-expression, it is interesting to note that vitamin d receptor (VDR) gains high weight in the TF-binding model (with rs141329158 as the non-synonymous SNP), with reported interaction with HLA DRB1 in type 1 diabetes patients from North India [70].

### 3.4. Explanatory Trans Associations Display Tissue-Specific Regulation of Immune Response

We define explanatory trans associations as hit genes where our TF models explain at least the same portion of the gene expression (in terms of R^2^) as the cis-based model. There were 787 hit genes (76%) with explanatory trans associations (Table S1). In total, 36 (3.5%) of them were discovered in both tissues, and an additional 9 hit genes are explanatory trans associations in one tissue but not in another.

The hit genes with the highest explanatory trans associations are transporter 1, ATP binding cassette subfamily B member (TAP1), with R^2^ = 0.63 in whole blood and R^2^ = 0.31 in skeletal muscle (PrediXcan R^2^ = 0.02 in both tissues). Regulation of the TAP1 gene is critical for the initiation and continuation of a cellular immune response [71]. Furthermore, STAT1 is necessary for the activation of TAP1 gene expression and the initiation of cellular immune responses [71], where STAT1 is the TF with the highest weight in our TF model in both tissues (weights = 0.33 and 0.29 in blood and muscle, respectively). Another TF with a high weight in both tissues is IRF1 (weights of 0.16 and 0.22 in blood and muscle, respectively), where regulation of TAP1 by IRF1 explains the deficiency of CD8+ t cells in IRF1 deficient mice [72].

Another top hit gene with the highest explanatory trans associations in whole blood is interferon regulatory factor 9 (IRF9) (R^2^ = 0.63, where PrediXcan R^2^ is only 0.01). The TFs with the highest weights in the IRF9 model are STAT1 (β = 0.28) and STAT2 (β = 0.24). Supporting their large weight in the model, the signaling cascade activated by type I and type III interferons is dominated by the formation of a complex comprising IRF9, STAT1, and STAT2 [73,74,75]. Type I and III interferons are known to promote anti-viral immune responses [76], with type III also specifically with the blood–brain barrier [77] and regulator of neutrophil function [78]. While IRF9 is integral in mediating the type I interferon anti-viral response and the role of IRF9 in many important non-communicable diseases has just begun to emerge, the regulation of IRF9 during these conditions is not well understood [79]. What is known is that high levels of IRF9 and STAT1/STAT2 drive a prolonged response of the initial anti-viral response and also provide resistance to DNA damage [80,81].

IRF9 and STAT1 play a role in the pathogenesis of systemic lupus erythematosus [82,83], which was previously mentioned to be highly enriched with the blood TF-expression model hit genes. The second top hit gene with a major trans effect in whole blood is interferon-induced protein with tetratricopeptide repeats 3 (IFIT3) (R^2^ = 0.47) with major trans effects but lower variability explained in skeletal muscle (R^2^ = 0.11). Abnormal elevations in IFIT3 are associated with stimulants of interferon genes signaling in human systemic lupus erythematosus monocytes [84,85]. While the highest-weighted TF in the IFIT3 model is IRF9 in both tissues (weights = 0.48 and 0.16, in blood and muscle tissues), STAT1 and STAT2 have much higher weights in blood (weights = 0.17 for both) in blood but negligible ones in muscle, suggesting a tissue-specific regulation of IFIT3.

## 4. Discussion

We hypothesized that variability in TFs may be associated with variability of transcription levels in their transcribed genes. We tested our hypothesis using two types of TF models, based on proposed mechanisms for the effect—a model assuming that change in the expression levels of the TF affects the expression levels of the gene, and a model considering the effect of change in binding affinity between TF and TF-binding site.

Our focus here was on describing the methodology, and we thus demonstrated it on the selected set of tissues. Sample size can affect the performance of gene imputation [86]; thus, we selected the tissues with the largest sample size in GTEx, enabling us to test the robustness of the results by repeating the analysis on sub-samples of the data. We further passed our models through rigorous background model tests to reduce type I errors.

Our work suggests that the discovered hit genes are tissue specific, which corresponds well to the previous observation of Lopes-Ramos et al. [87] that TF regulation is tissue specific, including observations in the whole-blood tissue, used in our study. Additionally, there are different genes discovered in the TF-expression and the TF-binding models, suggesting that these mechanisms may be complementary.

Based on the TF-binding results, polymorphism within TFs affects a smaller fraction of genes than with TF-expression models. This result may be explained by the complexity of the regulatory plan. As TFs typically transcribe several genes, major changes in TF binding or regulation may result in significant effects on several genes and cellular processes, which could be detrimental to the individual. Such genetic variation may suffer from evolutionary pressure to attenuate its effect. Given the enrichment of immune system hits in our TF-expression models, we could speculate that in the case of the immune system, a complex regulation plan that is not “hard-coded” into the genome but rather regulated on the transcription level might provide more benefits. This immune-specific regulation complexity was previously discussed, but more research is required to quantify it [88,89].

The lower number of hit genes identified by the TF-binding model than hit genes identified by the TF-expression model could potentially be a result of our focus on deleterious non-synonymous SNPs. There are two reasons why we focused on these SNPs. The first reason concerns the biological interpretability of our model. As we built this model under the assumption that effects on gene expression are through altered binding affinity, variants predicted to be deleterious would be the initial suspects, as they are expected to have the largest effect on the TF conformation and ultimately the binding affinity. The second reason is that incorporating a more focused set of variants helps in avoiding overfitting the models in the high-dimensional sparse space of variants. Thus, our detected hit genes from the TF-binding model could be regarded as an underestimate of the *trans* effect of variants within TFs.

While we propose a hypothesis-driven approach, our findings only establish associations and not causation. While further validation is required to establish causal relationships, our findings can be useful in prioritizing genes and TFs for experimental validation.

## 5. Conclusions

Understanding how variations in TFs regulate gene expression can offer key insights into the transcriptional regulation plan. Here, we demonstrated how we identify expression variations of TFs and combinations of SNPs within TFs that associate with gene expression levels, some with potential associations with diseases. Our models can help in two ways. The first way is by introducing new factors that can be associated with human phenotypes in large association studies, potentially improving phenotype prediction models. The second way is by generating new hypotheses for genetic architecture that can be further explored in additional experiments. Thus, our models should be integrated into future gene expression imputation methods, allowing us to improve association studies and gain a better understanding of human genetic architecture.

## Figures and Tables

**Table 1:** Summary statistics of input data per tissue: transcription factors and associated SNPs.

	Skeletal Muscle	Whole Blood
Number of samples	706	670
Number of expressed genes	21,031	20,315
Number of expressed genes with an associated TF (% of genes)	11,130 (53%)	10,563 (52%)
Number TFs per gene	10.8 ± 8.4	10.9 ± 8.7
Number of nsSNP * per TF	1.55 ± 1.77	1.57 ± 1.99

* nsSNP: non-synonymous SNP.

## Data Availability

GTEx data are available from dbGaP (Accession Number: phs000424.v7.p2). Transcription factors and their transcribed genes are publicly available from Transcriptional Regulatory Relationships Unraveled by Sentence-based Text Mining (TRRUST V2) [12], the Human Transcriptional Regulation Interaction Database (HTRI) [13], and the Regulatory Network Repository of Transcription Factor and microRNA Mediated Gene Regulations (RegNetwork) [14]. Code is freely available through GitHub: [90].

## Supplementary Materials

The following supporting information can be downloaded at: https://www.mdpi.com/article/10.3390/genes13050929/s1, Table S1: Hit genes and their corresponding R^2^ performance for the skeletal muscle and whole-blood TF-expression and TF-binding models. Table S2: Hit genes and their corresponding TFs with their LASSO model weights for the skeletal muscle and whole blood TF-expression and TF-binding models. Table S3: Model weights for TFs in TF-Expression model and nsSNPs for the TF-Binding model for all the hit genes.
